# Overcoming non-patient and health professional’s barriers to COVID-19 vaccination in pregnancy

**DOI:** 10.7189/jogh.12.03010

**Published:** 2022-03-19

**Authors:** Emmanuel Lamptey

**Affiliations:** Institute of Life and Earth Sciences, Pan African University, University of Ibadan, Ibadan, Nigeria

COVID-19 vaccines are recommended in pregnancy to protect against known risk of the infection, including admission to intensive care and premature birth of the baby [1]. Growing evidence about the safety and effectiveness of COVID-19 vaccines during pregnancy suggests that the benefits of receiving them outweigh any known or potential risks and or cause fertility problems in women [2].

That is the current recommendation of the Centers for Disease Control and Prevention (CDC) [2]. The WHO has stated they do not have any reasons to believe there will be a specific risk that would outweigh the benefits because pregnant women are at high risk of exposure to SARS-CoV-2. COVID-19 vaccines are approved for use by pregnant women, those planning pregnancy and breastfeeding mothers [3]. Immunization is commended in consultation with health care providers, and the decision to have the vaccine is a personal choice. Vaccination efforts and compliance are met with mixed feelings, repeated arguments, hesitancy, and there are still many people who fear the vaccines [4].

It has created a major impediment to a comprehensive vaccine roll-out, and emerging patients and health providers barriers to COVID-19 vaccination of the pregnant population exist. Besides these barriers, other challenges occur outside the scope of patients and their caregivers, which must be identified, tackled and facilitators enhanced. This paper sought to identify those barriers and ways of overcoming them with emphasis on different countries and contexts from a review of recent published literature on the topic.

## BARRIERS EXISTING IN DIFFERENT COUNTRIES AND CONTEXTS

The headline barrier overshadowing that of patients and health care providers remained the lack of safety data surrounding COVID-19 vaccination in pregnancy. Globally, countries have different policies on COVID-19 vaccination in pregnancy. As of 28 June 2021, 41 nations do not recommend the vaccine, 17 have not accepted it yet but with exemptions, and 29 only permitted it with qualifications [5].

India initially excluded pregnant women from the vaccines due to government concerns about safety issues [6]. In Germany, the standing committee on COVID-19 vaccination in pregnant women recommended the vaccines only in some individual cases, reasoning that there are not enough data to support the vaccination of all pregnant women [7]. Pregnant women being able to access COVID-19 vaccines depends on their location [8].

The variability in position or policies is due to insufficient evidence on vaccines in pregnancy or the exemption of pregnant and lactating women from clinical trials [9]. Many countries split in their policy response because they do not have data speaking specifically to this question [8].

Amid safety concerns, there are low prioritization of pregnant women, even though they are known to be at a significantly high risk of severe COVID-19 related complication [10]. Pregnant women are not solely prioritized because COVID-19 related deaths happen among old aged and those with co-morbidity [11]. Vaccine supplies have been directed to meet the needs of this population rather than pregnant women [11].

India and Indonesia are among the countries affected by high COVID-related maternal and under-five mortality as they have excluded pregnant women as a priority group [12].

In most countries, immunization campaigns are focused on priority groups because there are not enough supplies for pregnant women [11]. Some wealthier countries also secure as many vaccines as possible affecting supplies and accessibility elsewhere [13]. In some situations, richer countries have donated limited vaccine supplies to low and middle-income countries resulting in these countries receiving vaccines from different manufacturers and with varying preparation methods.

Unlike developed countries, there are no well-defined channels in place for testing, approving and regulating vaccines causing confusion and apprehension in an already vaccine hesitant pregnant population [11]. Receiving donations from multiple sources makes independent safety assessment more complex, and some countries declare or limit preference for certain vaccines due to their independent assessment of vaccine effects and safety profiles [11].

Still, on why more expectant mothers are not getting the lifesaving shots, the overarching cultural attitude of pregnancy plays a key role. In many contexts, the history of pregnancy revolves around the fact that pregnant women are to stay void of external influences or pregnancy is a fragile state that must be kept pure from pollution [14]. Some Pregnant women are worried and feel unsafe injecting a vaccine or foreign substance into their bodies, and this historical theory cut across the globe [14]. They opt for what feels safe rather than what is safe [14].

**Figure:**
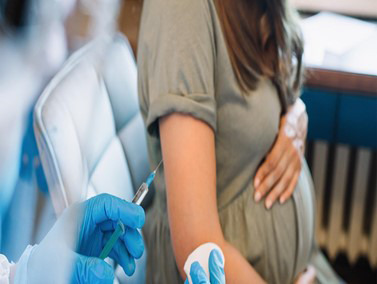
Photo: Source: NYU Langone Health.

To overcome these barriers, all individual countries must define, implement and recommend COVID-19 vaccination in pregnancy based on evaluation of the available scientific evidence and the best practices at the international level. Scientific evidence shows that the COVID-19 vaccines are safe for pregnant women, and these safety profiles should be reviewed regularly based on emerging dynamic evidence. For instance, Expectant mothers in India can now opt for a COVID-19 jab after the government revised its position regarding vaccinations for pregnant women [15]. Countries like South Africa and Bangladesh are coming forth in that direction to include all pregnant and reproductive-age women for COVID-19 vaccination [11].

In many countries, most of the health workers are females who are either pregnant or breastfeeding, targeting these populations will partly reduce the threat in the high-risk group and provide a gender-inclusive response [9]. Successful population-based inoculation against COVID-19 means pregnant and lactating women must be included [8]. In Mexico, pregnant women are prioritized for vaccines, and not a single vaccinated woman has died of COVID-19 during pregnancy [16].

In addressing issues of limited supplies, low and middle-income countries should collaborate with international stakeholders to expand their COVID-19 vaccine production capacity [17]. This initiative will resolve the unavailability of vaccines to help focus and prioritize vaccine distribution on the current and future benefits of pregnant women and their unborn children [18].

Developing countries and their donor communities can leverage joint procurement mechanisms and voluntary or compulsory licensing agreements for equitable and affordable access of the vaccines to pregnant women [19]. In cases of time supply shortages and mixed decisions about which group to prioritize for access, scaling up the production capacity of COVID-19 vaccines in these countries is vital for solving these challenges [18,20].

For government mistrust, mixed messaging and its conspiracy theories among the pregnant population, community-based care that builds confidence and trust must be deployed. The decision to accept or decline the jabs should be shared. Pregnant women should be provided with a balanced and clear assessment of their risk of COVID-19 in pregnancy, considering their circumstances, as well as local and international practices. Available scientific evidence must be presented, and patients express their values based on their concerns and autonomy [14]. To the extent that all fears, expectations, and worries are fully disclosed. The most important ones should be addressed and prioritized by their authorized caregiver through patient-centered communication.

By this approach, many who are skeptical can be convinced to get vaccinated in the pregnant population [14].

## CONCLUSIONS

Pregnant women are at higher risk of complications from COVID-19 infections. Immunization is the most effective intervention to mitigate the burden of SARS-CoV-2 in pregnancy.

Besides patient and health providers’ barriers, countries face other challenges - these have been identified and discussed above. To optimize COVID-19 vaccination in pregnancy, these barriers must be recognized and addressed. All countries should take various positions to maximize access to this population.
